# Silent Septic Arthritis: A Case Report

**DOI:** 10.7759/cureus.13579

**Published:** 2021-02-26

**Authors:** Amr Mohamed

**Affiliations:** 1 Internal Medicine, Rochester Regional Health, Rochester, USA

**Keywords:** case report, streptococcal bacteremia, septic arthritis

## Abstract

Patients with septic arthritis are observed regularly in daily hospital practice, and most patients have a clearly confirmed diagnosis. Here, we present a patient with poorly controlled diabetes mellitus in silent septic shock. Following careful clinical examination and identification of the infection source, the culprit lesion was drained. This case report highlights a history of falls as a sign of silent septic arthritis. Diagnostic knee taps should be performed when silent sepsis is suspected as clinical symptoms may be masked and not always be discernable in patients with poorly controlled diabetes.

## Introduction

Septic arthritis involves infection of a joint by an organism, frequently presenting as a swollen, extremely tender joint associated with sepsis criteria, including fever and leukocytosis [1]. Typically, a patient mainly complains of feeling very unwell; however, this is not always the case among patients with poorly controlled diabetes mellitus (DM) and among older adult patients in whom an atypical presentation can occur [2]. We describe a patient with a history of poorly controlled DM and a recent history of falls, who was afebrile, with normal vital signs on presentation, and who first presented with minimal complaints. He subsequently progressed to septic shock with high-grade streptococcal bacteremia. After a careful clinical examination, he was diagnosed with septic arthritis.

## Case presentation

**Figure 1:**
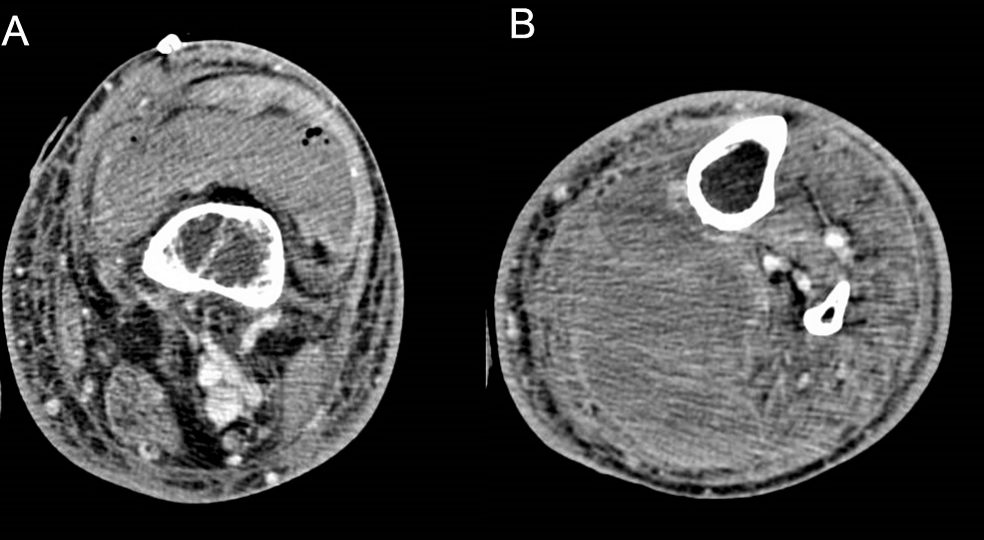
(A) Suprapatellar effusion with hyperdensity representing undrained infection. There are also scattered foci of air within the joint fluid likely representing postoperative changes. (B) Large complex lobulated hyperdense interfascial collection within the posterior knee/leg measuring up to 17.5 x 6.8 x 7.3 cm.

A 51-year-old man with a medical history of end-stage renal disease and poorly controlled DM was admitted to the hospital after syncope with head trauma. His neurological and cardiac workups were negative and his physical examination was within normal limits except for a slightly tender and mildly swollen left knee, which was attributed to his fall. The patient went into septic shock and was started on norepinephrine for vasopressor support. Blood culture results were Streptococcus dysgalactiae positive, and he was started on ceftriaxone. However, the infection source remained unclear. As his left knee swelling had increased compared to baseline on admission, a diagnostic tap was performed that showed purulent fluid comprising 250,000 white blood cells, which were predominantly neutrophils. Our patient underwent surgical drainage in the operating room (OR), where 100 mL of purulent fluid was drained from his left knee; however, he remained in a state of sepsis and was continued on vasopressors. We undertook a computed tomography (CT) scan of the left knee as shown in Figures 1A, 1B, which showed infection extending into the popliteal fossa, along with compression of the popliteal artery and vein. He underwent further surgery to drain 500 mL of purulent fluid from the popliteal fossa.

Cultured samples taken from the knee joint positively confirmed Streptococcus, which was consistent with his blood sample results. Given the extent of the infection, and because he was in septic shock with high-grade bacteremia, we administered a synergistic dose of gentamycin, and management was guided by infectious disease consultants.

Postoperatively, his condition improved, vasopressors were weaned, and a transthoracic echocardiogram was performed to rule out infective endocarditis, the results of which were found to be vegetation negative. Therefore, we did not proceed to perform transesophageal echocardiography as the treatment duration was four weeks. He remained on ceftriaxone for four weeks, along with a synergetic dose of gentamycin with dialysis, and he was followed up in an outpatient clinic and showed good progress.

## Discussion

Septic arthritis typically presents as a single, warm, swollen, and painful joint with restricted mobility [1]. The knee has been reported to be the involved joint in >50% of patients [2], and patients present as febrile and in sepsis. However, older adults and poorly controlled patients with DM may present as afebrile and asymptomatic [3].

The usual causative organism is Staphylococcus aureus (S. aureus) and infection is usually monomicrobial [4]; however, Streptococci are also an important cause of septic arthritis, although not as common [5]. Diagnosis is established based on clinical suspicion and through performing a diagnostic tap of the joint, typically we see purulent fluid with between 50,000 and 150,000 cells/µL of predominantly neutrophils in patients with septic arthritis [6].

Patient management involves joint drainage and empiric antibiotic therapy initially, which can later be narrowed according to laboratory findings [7]. The optimal duration of antibiotic treatment remains unclear [4]. Treatment recommendations have been described mainly for S. aureus. In this patient, we followed those recommendations, which state: “In the setting of bacteremia but without evidence of infective endocarditis, parenteral therapy is usually prescribed for four weeks; however, if there is no evidence of bacteremia or endocarditis, then two weeks of parenteral therapy followed by two weeks of oral therapy is sufficient. However, the decision is made based on the organism sensitivity pattern and clinical assessment" [4]. 

## Conclusions

Falls can be a sign of silent sepsis and diagnostic knee taps should be performed in suspected cases of septic arthritis as symptoms are not always discernable within a setting of poorly controlled DM.
